# Genetic relatedness between Japanese and European isolates of *Clostridium difficile* originating from piglets and their risk associated with human health

**DOI:** 10.3389/fmicb.2014.00513

**Published:** 2014-10-08

**Authors:** Masaru Usui, Yukie Nanbu, Kentaro Oka, Motomichi Takahashi, Takashi Inamatsu, Tetsuo Asai, Shigeru Kamiya, Yutaka Tamura

**Affiliations:** ^1^Laboratory of Food Microbiology and Food Safety, Department of Health and Environmental Sciences, School of Veterinary Medicine, Rakuno Gakuen UniversityHokkaido, Japan; ^2^Miyarisan Pharmaceutical Co., Ltd.Tokyo, Japan; ^3^Department of Infectious Diseases, Kyorin University School of MedicineTokyo, Japan; ^4^Department of Infectious Diseases, Tokyo Metropolitan Geriatric HospitalTokyo, Japan; ^5^The United Graduate School of Veterinary Sciences, Gifu UniversityGifu, Japan

**Keywords:** antimicrobial resistance, *Clostridium difficile*, MLVA, PCR ribotype 078, piglets

## Abstract

*Clostridium difficile* colonization in pig intestine has been a public health concern. We analyzed *C. difficile* prevalence among piglets in Japan to clarify their origin and extent of the associated risk by using molecular and microbiological methods for both swine and human clinical isolates and foreign isolates. *C. difficile* was isolated from 120 neonatal piglet fecal samples. Toxin gene profile, antimicrobial susceptibilities, PCR ribotype, and multiple-locus variable-number tandem-repeat analysis (MLVA) type of swine isolates were determined and compared with those of human clinical and foreign isolates. One-hundred *C. difficile* strains were isolated from 69 (57.5%) samples, and 61 isolates (61%) were toxin gene-positive. Some isolates were resistant to antimicrobials, contributing to antibiotic-associated diarrhea by *C. difficile*. These results suggest that *C. difficile*, prevalent among Japanese pigs, is a potential risk for antibiotic-associated diarrhea. Furthermore, PCR ribotype 078 (12 isolates), which has been linked to multiple outbreaks worldwide, was the third-most frequently isolated of the 14 PCR ribotypes identified. Moreover, MLVA revealed that all 12 PCR ribotype 078 isolates were genetically related to European PCR ribotype 078 strains found in both humans and pigs. To date, in Japan, many breeding pigs have been imported from European countries. The genetic relatedness of *C. difficile* isolates of Japanese swine origin to those of European origin suggests that they were introduced into Japan via imported pigs.

## INTRODUCTION

*Clostridium difficile* is a gram-positive spore-forming anaerobic bacterium that causes antibiotic-associated diarrhea and pseudomembranous colitis in humans. Most pathogenic *C. difficile* strains produce two structurally similar toxins, toxin A, and toxin B, the main virulence determinants associated with *C. difficile* infection. *C. difficile* is often isolated from patients and food-producing animals (Keessen et al., 2011). Although zoonotic transmission remains speculative, meat products could be a common source of *C. difficile* infection in humans, and food-producing animals could also serve as a reservoir.

In the past decade, *C. difficile* infections have become more common and more severe in developed countries, including Japan (Rupnik et al., 2009; Honda et al., 2014). Recent reports have demonstrated that the increase in *C. difficile* burden has been driven by a rapid change in the global epidemiology with the emergence of an epidemic strain of *C. difficile*. The strain PCR ribotype 027 (BI/NAP1) was detected initially in North America, and was observed subsequently in European countries, Australia, and some Asian countries (Richards et al., 2011; Collins et al., 2013; Knight et al., 2013). In addition, the prevalence of PCR ribotype 078, a strain commonly found in pigs, has increased since 2006 and is currently one of the most common types in European countries (Bauer et al., 2011). Some reports have highlighted the high-level relatedness that exists between *C. difficile* PCR ribotype 078 isolates from both human and swine origins by using highly discriminative typing methods such as multiple-locus variable-number tandem-repeat analysis (MLVA; Debast et al., 2009; Bakker et al., 2010). In Japan, PCR ribotype 027 has only been observed in sporadic cases and PCR ribotype 078 has not been detected, unlike in Europe and North America (Collins et al., 2013). PCR ribotype 018 (smz) is the most prevalent isolate detected from human cases of *C. difficile* infection in Japan (Collins et al., 2013). To evaluate the risk to human health, it is important to determine bacterial properties of swine *C. difficile* isolates, such as toxigenicity, genotype, and antimicrobial susceptibility, and compare them with those of human isolates.

The purpose of this study was to clarify the risk to human health associated with *C. difficile* isolates present in the swine population of Japan. We have previously attempted to isolate *C. difficile* from 250 slaughter pigs; however, *C. difficile* was isolated from only two fecal samples (Asai et al., 2013). *C. difficile* is more frequently isolated from neonatal piglets, rather than slaughter pigs (Hopman et al., 2011). In addition, there is no clear link between *C. difficile* isolation and neonatal porcine diarrhea (Alvarez-Perez et al., 2009). Therefore, we isolated *C. difficile* from clinically normal piglets to elucidate the present status of *C. difficile* in Japan. We assessed the toxigenicity and antimicrobial susceptibility of the *C. difficile* isolates, and determined the relatedness of *C. difficile* that originated from piglets, human clinical isolates, and foreign isolates by analyzing their molecular characteristics.

## MATERIALS AND METHODS

### BACTERIAL STRAINS AND FECAL SAMPLES

A total of 120 fecal specimens were collected from neonatal piglets less than 20 days of age. These samples were collected at 12 farms (10 piglets from different farrowing sows/farm) during June–August 2012 in seven different prefectures of Japan. The piglets did not exhibit symptoms of diarrhea at the time of sampling.

Two isolates of *C. difficile* obtained from slaughter pigs in Japan (Asai et al., 2013) were used in this study. Seventy-three clinical isolates of *C. difficile* that were obtained between 2002 and 2005 at two Tokyo hospitals (Oka et al., 2012) were also used in this study to compare their antimicrobial susceptibilities and genetic relatedness with the swine isolates. *C. difficile* strains 9689, 43593, 700057, BAA–1870, and BAA–1875 were obtained from the American Type Culture Collection (ATCC; Manassas, VA, USA) to serve as reference strains.

### CULTURE MEDIA

*Clostridium difficile* isolated from fecal samples that were treated with alcohol for spore selection as described previously (Asai et al., 2013), was cultured on cycloserine-cefoxitin-mannitol agar (CCMA)-Ex (Nissui Pharmaceutical, Tokyo, Japan) at 37^∘^C for 36–48 h under anaerobic conditions. Isolated colonies were purified by restreaking onto CCMA-Ex followed by anaerobic incubation as indicated above. For each fecal sample, a maximum of three colonies were identified as *C. difficile* based on colony morphology, and were then analyzed further.

### IDENTIFICATION AND TOXIN GENE DETECTION

DNA was extracted using a commercial kit (InstaGene Matrix, BioRad, Hercules, CA, USA) according to the manufacturer’s instructions. Polymerase chain reaction (PCR) with a specific primer set (**Table 1**; Kikuchi et al., 2002) was used to confirm bacterial identification. The presence of genes encoding toxin A, B, and binary toxin (*tcdA, tcdB*, and *cdtA/B*, respectively) was analyzed using multiplex PCR as described previously (Persson et al., 2008). Primer sequences are listed in **Table 1**. To investigate the 3′ end deletion in *tcdA*, a supplemental PCR was performed as described previously (Kato et al., 1999) using the primers (**Table 1**).

**Table 1:** Primers used in the present study.

Gene target	Primer name	Primer sequence (5′-3′)	Amplicon size (bp)	Reference
16S rDNA	CIDIF-F	CTTGAATATCAAAGGTGAGCCA	1085	Kikuchi et al. (2002)
	CIDIF-R	CTACAATCCGAACTGAGAGTA		
*tcdA*	tcdA-F3345	GCATGATAAGGCAACTTCAGTGGTA	629	Persson et al. (2008)
	tcdA-R3969	AGTTCCTCCTGCTCCATCAAATG		
*tcdB*	tcdB-F5670	CCAAARTGGAGTGTTACAAACAGGTG	410	Persson et al. (2008)
	tcdB-R6079A	GCATTTCTCCATTCTCAGCAAAGTA		
	tcdB-R6079B	GCATTTCTCCGTTTTCAGCAAAGTA		
*cdtA*	cdtA-F739A	GGGAAGCACTATATTAAAGCAGAAGC	221	Persson et al. (2008)
	cdtA-F739B	GGGAAACATTATATTAAAGCAGAAGC		
	cdtA-R958	CTGGGTTAGGATTATTTACTGGACCA		
*cdtB*	cdtB-F617	TTGACCCAAAGTTGATGTCTGATTG	262	Persson et al. (2008)
	cdtB-R878	CGGATCTCTTGCTTCAGTCTTTATAG		
*tcdA* 3′-end deletions	NK9	CCACCAGCTGCAGCCATA	2355^a^	Kato et al. (1999)
	NKV011	TTTTGATCCTATAGAATCTAACTTAGTAAC		


^a^Amplicon size when no deletion was present.

### PCR RIBOTYPING

Polymerase chain reaction ribotyping was performed as described previously (Stubbs et al., 1999; Oka et al., 2012). Briefly, the volume of the PCR mixture was downscaled from 50 to 15 μL, and the amplified PCR products were concentrated to a final volume of approximately 10 μL by heating at 75^∘^C for 90–120 min. Electrophoresis in 3% Metaphor agarose (Lonza Rockland Inc., Basel, Switzerland) at a constant voltage of 120 V for 4 h was used to separate the PCR products. The PCR ribotyping banding patterns were analyzed using the BioNumerics program (Applied Maths, Sint-Martens-Latem, Belgium). Similarity and diversity were assessed by applying the Dice coefficient. Cluster analysis was performed using the Unweighted Pair Group Method with Arithmetic Mean algorithm.

### ANTIMICROBIAL SUSCEPTIBILITY TESTING

We performed minimal inhibitory concentration (MIC) determinations using the agar dilution method according to the Clinical Laboratory Standards Institute [CLSI] (2007) guidelines. Susceptibility to vancomycin, metronidazole, clindamycin, ceftriaxone, erythromycin, and ciprofloxacin (Sigma-Aldrich, St. Louis, MO, USA) was tested. The resistance breakpoints of metronidazole, clindamycin, and ceftriaxone adopted were those defined by the CLSI guidelines (Clinical Laboratory Standards Institute [CLSI], 2007). The breakpoints for vancomycin, erythromycin, and ciprofloxacin, which are not defined by the CLSI guidelines, were chosen as described in a previous report Oka et al. (2012). *C. difficile* ATCC700057 was used as a quality control strain. Antimicrobial susceptibility of 73 human clinical isolates of *C. difficile* was determined by the *E*-test Oka et al. (2012) and the agar dilution method to compare the MICs of swine and human clinical isolates.

### CHARACTERIZATION OF PCR RIBOTYPE 078 ISOLATES

The toxinotype of all PCR ribotype 078 isolates was determined by the method described by Rupnik et al. (2001). The *tcdC* sequence of all PCR ribotype 078 isolates was determined as described by Spigaglia and Mastrantonio (2002).

### MULTIPLE-LOCUS VARIABLE-NUMBER TANDEM-REPEAT ANALYSIS (MLVA)

The MLVA of all PCR ribotype 078 isolates was determined by the optimized MLVA method based on six loci (Bakker et al., 2010). PCR was carried out as described by Bakker et al. (2010). To confirm the number of tandem repeats, the PCR product was directly sequenced. The amplified product was purified with FastGene Gel/PCR Extraction Kit (Nippon Genetics, Tokyo, Japan) and sequenced in both directions using the same primers employed in the PCR. Nucleotide sequences were determined using the BigDye Terminator, version 3.1, Cycle sequencing kit with an automated DNA sequencer (ABI 3130; Applied Biosystems, Foster City, CA, USA). The number of tandem repeats at each locus was manually determined using the BioEdit software (). The motif copy numbers in the tandem array were imported into the BioNumerics software (Applied Maths) and a minimum-spanning tree was generated using the categorical coefficient of the software. We compared the MLVA profiles of our PCR ribotype 078 isolates and the foreign PCR ribotype 078 isolates derived from pigs and humans (Bakker et al., 2010).

## RESULTS

### ISOLATION OF *C. difficile* FROM PIGLET FECAL SAMPLES

Fecal samples from 120 piglets were analyzed, and *C. difficile* was isolated only from a subset of those piglets. When two or three isolates from a fecal sample exhibited the same PCR ribotype and antimicrobial susceptibility, they were considered to be a single isolate. Thirty-nine isolates were derived from 39 samples, 58 isolates were derived from 29 samples (two isolates from each sample), and three isolates were derived from one sample (three isolates from the single sample). In total, 100 *C. difficile* isolates were identified. *C. difficile* was isolated from 69 (57.5%) of the 120 samples obtained from 11 (91.7%) of the 12 farms (**Table 2**).

**Table 2:** Isolation and toxin gene profile of *Clostridium difficile* from Japanese piglets.

Farm	Location of prefecture	Samples	No. of positive samples	No. of isolates	Toxin A^+^B^+^		Toxin A^-^B^-^CDT^-^
					CDT^+^	CDT^-^	
A	Hokkaido	10	9	11	0	10	1
B	Hokkaido	10	7	8	0	8	0
C	Hokkaido	10	8	10	10	0	0
D	Hokkaido	10	7	13	0	0	13
E	Hokkaido	10	9	18	0	17	1
F	Miyagi	10	0	0	0	0	0
G	Miyagi	10	7	10	5	0	5
H	Yamagata	10	3	3	0	0	3
I	Fukushima	10	5	7	0	0	7
J	Tochigi	10	4	4	4	0	0
K	Chiba	10	7	9	6	0	3
L	Gifu	10	3	7	1	0	6

Sub Total					26	35	

Total		120	69 (57.5%)	100	61 (61.0%)	39 (39.0%)

### TOXIN GENE PROFILE

The toxin profile of the *C. difficile* isolates was examined by PCR. Of the 100 isolates of *C. difficile*, 61.0% (61/100) were positive for *tcdA* and *tcdB* (Toxin A^+^B^+^), among which 42.6% (26/61) were also positive for the binary toxin genes (*cdtA* and *cdtB*; CDT^+^; **Table 2**). 3′ end deletion in *tcdA* was not detected in all 61 strains positive for *tcdA*. Thirty-nine isolates (39.0%) were negative for *tcdA*, *tcdB*, *cdtA*, and *cdtB* (Toxin A^-^B^-^CDT^-^). One isolate of *C. difficile* derived from a slaughter pig (12EN) was also positive for *tcdA* and *tcdB* (Toxin A^+^B^+^) by the multiplex PCR method (Persson et al., 2008), although the isolate was only positive for *tcdB*, as determined using the primer set described by Kato et al. (1998). A deletion in *tcdA* that was complementary the NK11 primer, which was used for amplification of *tcdA* gene in the previous report Asai et al. (2013), was observed. The isolate of 12EN was also positive for binary toxin genes. The other isolate derived from a slaughter pig (134C1) was negative for toxin A, B, and binary toxin (Toxin A^-^B^-^CDT^-^).

### ANTIMICROBIAL SUSCEPTIBILITY

MIC_50_s, MIC_90_s, and the range of MICs of vancomycin, metronidazole, clindamycin, ceftriaxone, erythromycin, and ciprofloxacin for the 100 *C. difficile* isolates were determined (**Table 3**). All isolates were susceptible to vancomycin and metronidazole. Resistance against clindamycin, ceftriaxone, erythromycin, and ciprofloxacin were found in 59, 6, 46, and 75% of the isolates, respectively. Of the 61 toxigenic *C. difficile* isolates (Toxin A^+^B^+^), the incidence of resistance to clindamycin, ceftriaxone, erythromycin, and ciprofloxacin was 71, 10, 43, and 74%, respectively. The percentage of resistant isolates derived from piglets against all antimicrobials, particularly ceftriaxone, was lower than that clinically isolated from humans (Oka et al., 2012).

**Table 3:** Antimicrobial susceptibility of *Clostridium difficile* piglet isolates and human clinical isolates.

		Piglet				Human^c^			
	MIC (μg/mL)	MIC (μg/mL)			No. of resistant isolates (*n* = 100)	MIC (μg/mL)			No. of resistant isolates (*n* = 73)
Antimicrobials	Break point	50%	90%	Range		50%	90%	Range	
Vancomycin	≥32^a^	1	2	1–4	0	0.5	1	0.06–2	0
Metronidazole	≥32^b^	0.5	8	0.125–8	0	0.25	0.25	<0.06–0.25	0
Clindamycin	≥8^b^	8	256	0.25–>256	59 (59.0%)	256	>256	0.125–>256	64 (87.7%)
Ceftriaxone	≥64^b^	16	32	2–>256	6 (6.0%)	256	256	0.125–>256	62 (84.9%)
Erythromycin	≥8^a^	2	>256	1–>256	46 (46.0%)	>256	>256	<0.125–>256	61 (83.6%)
Ciprofloxacin	≥4^a^	8	8	0.5–128	75 (75.0%)	64	64	0.125–64	68 (93.2%)

*MIC, minimum inhibitory concentration.*

*^a^The value was the previous report break point (Oka et al., 2012).*

*^b^The value was the CLSI break point (Clinical Laboratory Standards Institute [CLSI], 2007).*

*^c^These isolates were isolated in the previous report Oka et al. (2012).*

### GENOTYPING

**FIGURE 1:**
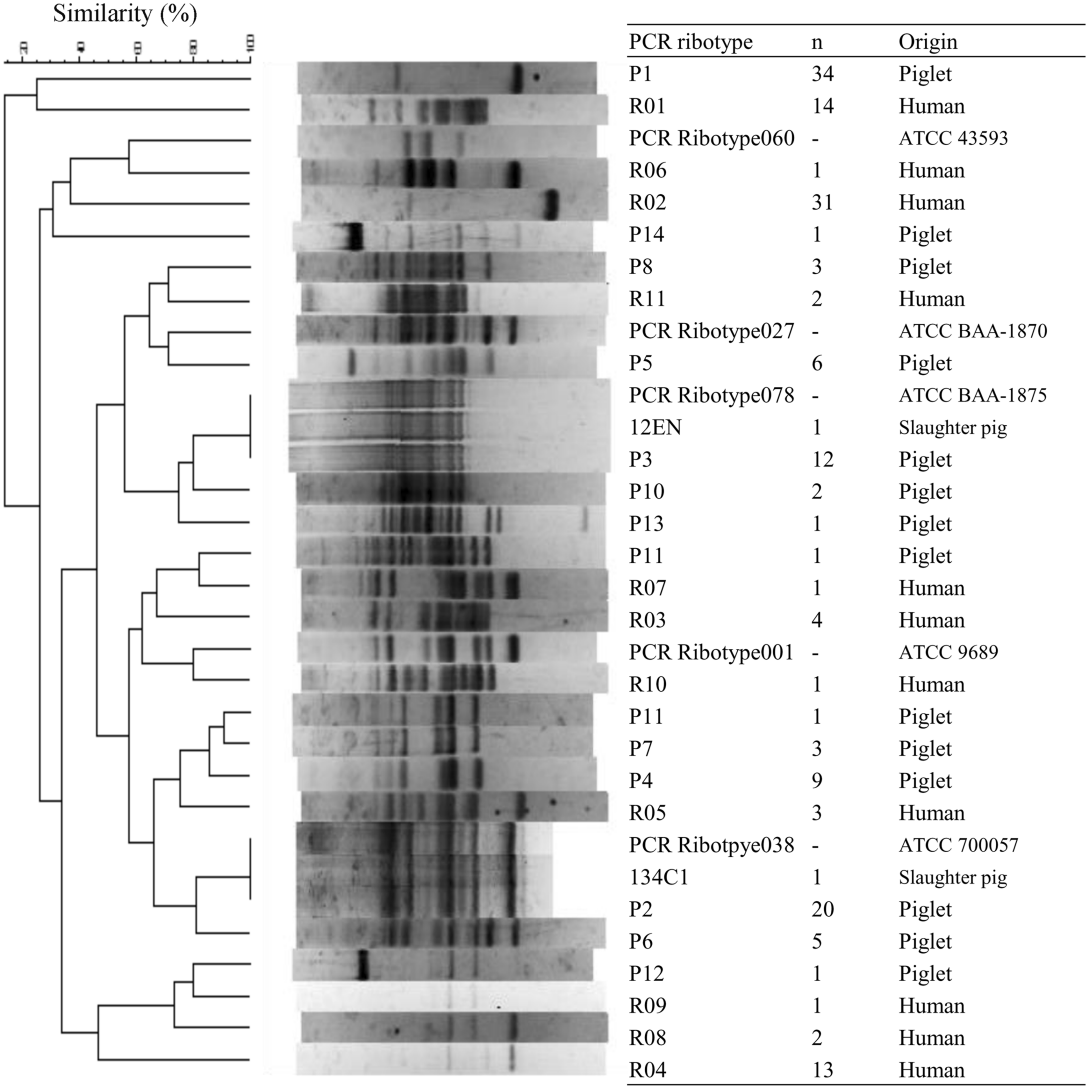
**Polymerase chain reaction (PCR) ribotype profiles of *Clostridium difficile* strains isolated from piglets, slaughter pigs, and humans.** PCR ribotyping pattern analysis was performed by applying the Dice coefficient. Sixty-nine isolates from piglets were isolated in this study. Two isolates from slaughter pigs and 73 isolates from humans were isolated previously (Oka et al., 2012; Asai et al., 2013). ATCC strains were used as control strains.

All 100 isolates were PCR ribotypeable and resolved into 14 PCR ribotypes (**Figure 1**; **Table 4**). The major PCR ribotypes of *C. difficile* isolates were P1 (34 isolates), P2 (20 isolates), and P3 (12 isolates). The banding patterns of PCR ribotype P2 and PCR ribotype P3 was identical to that of ATCC 700057 (PCR ribotype 038) and ATCC BAA-1875 (PCR ribotype 078), respectively. The banding patterns of 134C1 and 12EN, which were derived from slaughter pigs (Asai et al., 2013), were identical to PCR ribotype P2 and PCR ribotype P3, respectively. None of the PCR ribotypes of *C. difficile* isolates from piglets were identical to those from human clinical isolates in Japan (Oka et al., 2012) or the reference strains *C. difficile* ATCC 9689 (PCR ribotype 001), ATCC 43593 (PCR ribotype 060), and ATCC BAA-1870 (PCR ribotype 027).

**Table 4:** Relationship between the PCR ribotype and the toxin gene profile.

PCR Ribotypes	No. of isolates	Toxin A^+^B^+^		Toxin A^-^B^-^CDT^-^
		CDT^+^	CDT^-^	
P1	34	8	9	17
P2	20	0	0	20
P3	12	12	0	0
P4	9	0	9	0
P5	6	0	6	0
P6	5	5	0	0
P7	3	0	3	0
P8	3	0	3	0
P9	2	2	0	0
P10	2	0	2	0
P11	1	1	0	0
P12	1	0	1	0
P13	1	0	1	0
P14	1	0	1	0

### CHARACTERIZATION OF PCR RIBOTYPE 078 ISOLATES

All 12 *C. difficile* PCR ribotype 078 strains belonged to toxinotype V. All 12 *C. difficile* PCR ribotype 078 strains were found to contain a 39-base pair deletion in the toxin regulator gene (*tcdC*). One isolate of *C. difficile* PCR ribotype 078 derived from a slaughter pig (12EN) belonged to toxinotype VI. This isolate also contains a 39-base pair deletion in *tcdC*.

### MLVA

**FIGURE 2:**
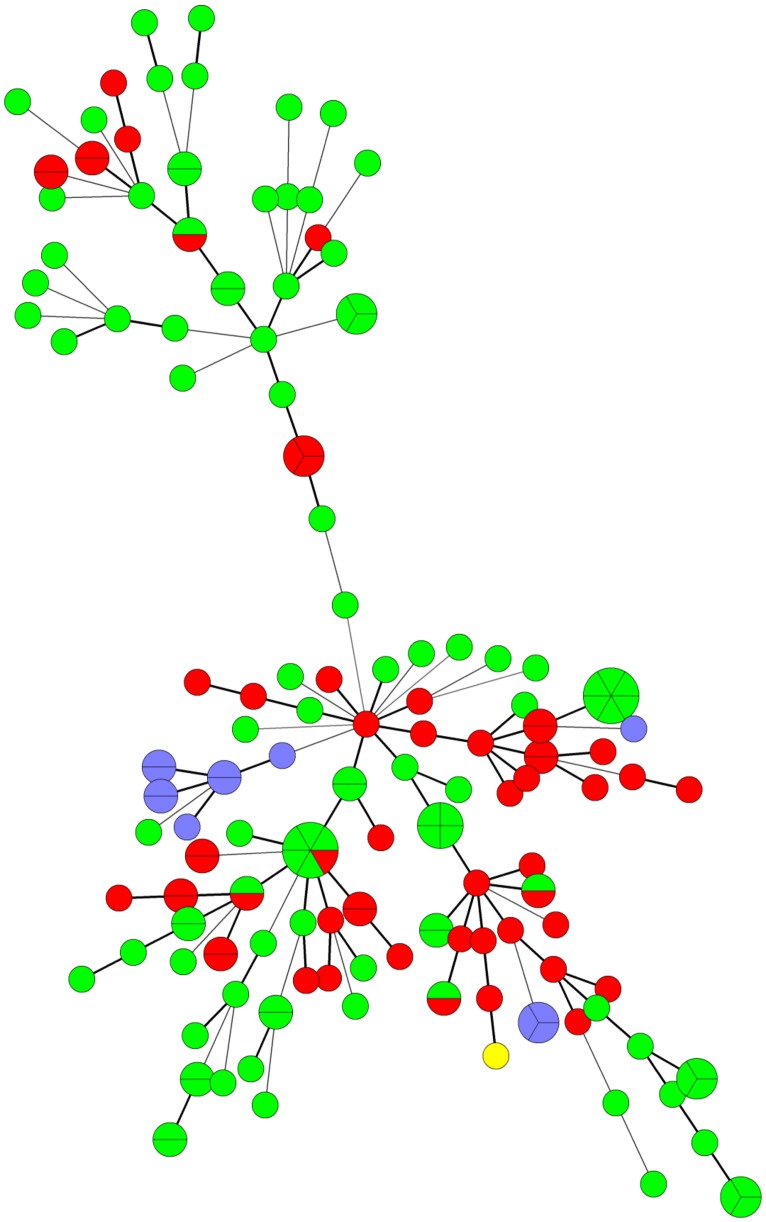
**Minimum spanning tree analysis of *C. difficile* PCR ribotype 078 isolates by multiple-locus variable number tandem repeat analysis (MLVA).** Each MLVA type is indicated by one node or branch tip, displayed as a circle that is connected by branches of minimum-spanning tree. The length of the branches represent genetic distances. The colors indicate the origin of isolates (European humans: green; European swine: red; Japanese piglet: purple; Japanese slaughter pig: yellow). European isolates derived from four European countries were investigated in the previous report Bakker et al. (2010).

In a previous report, 102 and 56 *C. difficile* PCR ribotype 078 strains of human and swine origins, respectively, from four European countries were investigated by MLVA (Bakker et al., 2010). The largest genetically related clusters (GCs) contained 103 strains, encompassing 47 swine strains and 56 human strains. In this study, all *C. difficile* PCR ribotype 078 strains from 12 piglets and one slaughter pig also belonged to the largest GCs (**Figure 2**; **Table 5**).

**Table 5:** For each strain, the results of the optimized MLVA for each of the 7 loci.

Isolate No.	Farm	MLVA result (No. of tandem repeats for indicated locus)						
		A6_cd_	B7_cd_	C6_cd_	E7_cd_	F3_cd_	G8_cd_	H9_cd_
1	E	NA	22	37	8	4	10	2
2	E	NA	22	37	8	4	10	2
3	E	NA	22	37	8	4	10	2
4	F	NA	8	36	9	4	4	2
5	J	NA	22	34	8	4	11	2
6	J	NA	21	36	8	4	11	2
7	J	NA	22	36	8	4	11	2
8	J	NA	22	35	8	4	11	2
9	J	NA	18	36	8	4	11	2
10	J	NA	22	35	8	4	11	2
11	J	NA	22	36	8	4	11	2
12	J	NA	21	36	8	4	11	2
12EN^a^		NA	25	33	8	4	12	2

*NA, not applicable.*

*^a^This isolate was derived from slaughter pig (Asai et al., 2013).*

## DISCUSSION

We demonstrated the high prevalence of *C. difficile* in clinically normal piglets (57.5%), despite the previous isolation of only two strains from slaughter pigs (0.8%) in Japan (Asai et al., 2013). Hopman et al. (2011) suggested that piglets acquire *C. difficile* shortly after birth. A previous study revealed that piglets become infected with *C. difficile* through contamination of the environment in the farrowing crates (Hopman et al., 2011). A significant reduction in the rate of colonization with *C. difficile* with age has also been reported Norman et al. (2009). The PCR ribotypes of two isolates from slaughter pigs (Asai et al., 2013) belonged to that of the two dominant PCR ribotypes detected among piglet strains (P2 and P3; **Figure 1**). Although the prevalence of *C. difficile* was relatively low in slaughter pigs, the presence of healthy pigs carrying this pathogen in slaughterhouses poses a significant potential for the contamination of meats and subsequent human infection (Weese et al., 2011).

The current study showed that several strains could be isolated from a single sample. In many cases, each of these isolates yielded different PCR ribotypes. These results suggest that various types of *C. difficile* might co-exist in the piglet intestine. The most prevalent PCR ribotype in this study was P1. This ribotype indicated several toxin gene profiles. Previously, one PCR ribotype indicated the same toxin gene profile, except for a rare case (Martin et al., 2008). The second most prevalent PCR ribotype P2 was then non-toxigenic PCR ribotype 038. The prevalence of this ribotype has not been reported in other countries in piglet. In European countries, PCR ribotype 078 derived from piglets was the most prevalent PCR ribotype (Keel et al., 2007; Hopman et al., 2011; Schneeberg et al., 2013). These results suggest that the distribution of this PCR ribotype in Japan was unique. Although the toxin gene profile of P1 was unusual, this ribotype should be monitored in Japanese pigs.

This study demonstrated that toxigenic *C. difficile* (Toxin A^+^B^+^) were isolated with high prevalence (61.0%) from piglets in Japan. The binary toxin genes, which contribute to the severity of infection (Bacci et al., 2011), were found in 26% of the isolates. Some *C. difficile* isolates were also resistant to the tested antimicrobials which are linked to antibiotic-associated diarrhea caused by *C. difficile* (Bartlett and Gerding, 2008). Comparing the antimicrobial susceptibility between piglet isolates and human clinical isolates, the resistance rate of piglet isolates was lower than that of human clinical isolates. These findings suggest that human clinical isolates are frequently exposed to the antimicrobials in clinical practices, resulting in a higher incidence of resistance. Moreover, the low percentage of ceftriaxone resistance (6.0%) in swine isolates is likely due to the rare usage of cephalosporin in pigs (Ministry of Agriculture Forestry and Fisheries [MAFF], 2012). The hypervirulent PCR ribotype 078 has caused serious outbreaks in humans worldwide and has been detected in both humans and pigs among European countries (Bakker et al., 2010; Keessen et al., 2011). Currently, PCR ribotype 078 is the third dominant PCR ribotype among Japanese piglets, although it has not been isolated in the human clinical setting in Japan (Collins et al., 2013). Our results indicate that piglets are potential reservoirs of toxigenic and antimicrobial-resistant *C. difficile*, including PCR ribotype 078, in Japan.

*Clostridium difficile* can survive in the environment for several months because of its spore-forming ability (Kim et al., 1981). Vermin can also play a role in the spread and transmission of *C. difficile* within pig farms and to other locations (Burt et al., 2012). In addition, as the manure of pigs, including piglets, are used to fertilize crops, *C. difficile* can pollute the soil and contaminate vegetables (al Saif and Brazier, 1996). Therefore, *C. difficile* can be readily transferred to humans via both animal products and vegetables. In addition, pigs are sometimes kept as pet. The pets are thought to pose a high risk transmission of *C. difficile* to humans, because of close contact with humans. In the current study, *C. difficile* was not isolated from farm F. Therefore, reducing *C. difficile* infection in piglets is possible. However, we could not clarify the reason for the negative test for *C. difficile* in farm F, and so a future study should assess the cause of this observation. To minimize the risk posed by *C. difficile*, it is necessary to develop effective hygiene management practices to aid in the limitation of the dissemination of *C. difficile*.

It is important to establish an epidemiological analysis for *C. difficile* to clarify its origin in pigs in Japan. MLVA revealed that swine isolates of PCR ribotype 078 in Japan were genetically related to European isolates from both humans and pigs (Bakker et al., 2010). In addition, Japanese isolates of PCR ribotype 078, except for one strain isolated from a slaughter pig (Debast et al., 2009), were toxinotype V and contained a 39-base pair deletion in the toxin regulator gene (*tcdC*), the same as European PCR ribotype 078 isolates (Pirs et al., 2008). Many breeding pigs have been imported to Japan from European and North American countries (Baba et al., 2010). In European countries, the isolation of PCR ribotype 078 from piglets was more prevalent compared with the current study (Keel et al., 2007; Hopman et al., 2011; Schneeberg et al., 2013). The spread of pathogenic bacteria such as methicillin-resistant *Staphylococcus aureus* could be related to the global distribution of the pigs (Espinosa-Gongora et al., 2012). These results raise the possibility that *C. difficile* PCR ribotype 078 was brought to Japan via the import of breeding pigs.

In conclusion, 14 PCR ribotypes of 100 *C. difficile* strains isolated from piglets were distinguishable from those of 73 human clinical isolates included in this study. As all human clinical isolates were isolated from only two hospitals in Tokyo, large-scale studies are essential to clarify the relatedness between human clinical isolates and animal isolates in Japan. This study revealed that *C. difficile*, prevalent among Japanese pigs, is a potential risk for antibiotic-associated diarrhea. Although PCR ribotype 078 were isolated in Japanese piglets, a unique distribution of PCR ribotypes was observed in Japan. Continuous surveillance of *C. difficile* PCR ribotype 078 among human clinical isolates is also necessary.

## Conflict of Interest Statement

The authors declare that the research was conducted in the absence of any commercial or financial relationships that could be construed as a potential conflict of interest.
